# 2-Chloro-4-(3,3-dichloro­all­yloxy)-1-nitro­benzene

**DOI:** 10.1107/S160053681201865X

**Published:** 2012-05-02

**Authors:** Xiao-feng Yu, Zheng-jun Xia, Chun-ya Li

**Affiliations:** a School of Pharmaceutics, Jiangsu University, Zhenjiang 212013, People’s Republic of China; b R&D Center, Jiangsu Yabang Pharmaceutical Group, Liangchang Road East No. 6 Jingtan, Changzhou 213200, People’s Republic of China

## Abstract

In the crystal structure of the title compound, C_9_H_6_Cl_3_NO_3_, mol­ecules are connected by C—H⋯O hydrogen bonds, forming chains along the *b* axis. The dihedral angle between the benzene ring and the plane of the nitro group is 16.2 (1)° and that between the benzene ring and the plane of the dichloro­allyl group is 10.2 (1)°.

## Related literature

For background to the applications of the title compound, see: Kolosov *et al.* (2002 ▶). For the synthesis, see: Walker *et al.* (2005 ▶).
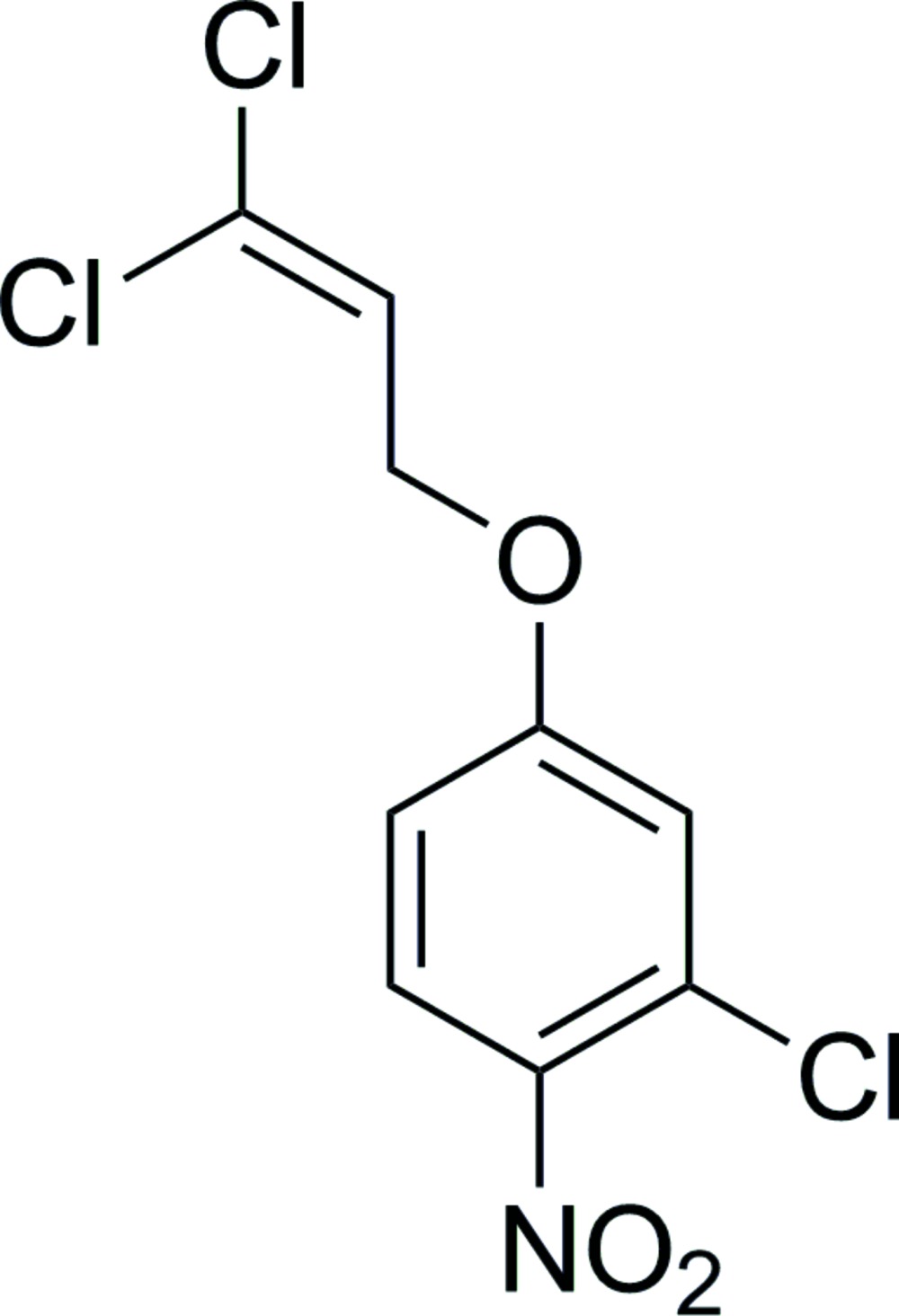


## Experimental

### 

#### Crystal data

C_9_H_6_Cl_3_NO_3_
*M* *_r_* = 282.50
Monoclinic, 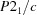

*a* = 12.476 (3) Å
*b* = 12.775 (3) Å
*c* = 7.2230 (14) Å
β = 92.32 (3)°
*V* = 1150.3 (4) Å^3^
*Z* = 4
Mo *K*α radiation
μ = 0.79 mm^−1^
*T* = 293 K
0.30 × 0.20 × 0.10 mm

#### Data collection

Enraf–Nonius CAD-4 diffractometer
Absorption correction: ψ scan (North *et al.*, 1968 ▶) *T*_min_ = 0.799, *T*_max_ = 0.926
2300 measured reflections
2118 independent reflections
1414 reflections with *I* > 2σ(*I*)
*R*_int_ = 0.023
3 standard reflections every 200 reflections intensity decay: 1%

#### Refinement

*R*[*F* ^2^ > 2σ(*F* ^2^)] = 0.066
*wR*(*F* ^2^) = 0.183
*S* = 1.00
2118 reflections
145 parameters
H-atom parameters constrained
Δρ_max_ = 0.52 e Å^−3^
Δρ_min_ = −0.42 e Å^−3^

### 

Data collection: *CAD-4 Software* (Enraf–Nonius, 1985 ▶); cell refinement: *CAD-4 Software*; data reduction: *XCAD4* (Harms & Wocadlo, 1995 ▶); program(s) used to solve structure: *SHELXS97* (Sheldrick, 2008 ▶); program(s) used to refine structure: *SHELXS97* (Sheldrick, 2008 ▶); molecular graphics: *SHELXTL* (Sheldrick, 2008 ▶); software used to prepare material for publication: *SHELXTL*.

## Supplementary Material

Crystal structure: contains datablock(s) I, global. DOI: 10.1107/S160053681201865X/vm2169sup1.cif

Structure factors: contains datablock(s) I. DOI: 10.1107/S160053681201865X/vm2169Isup2.hkl

Supplementary material file. DOI: 10.1107/S160053681201865X/vm2169Isup3.cml

Additional supplementary materials:  crystallographic information; 3D view; checkCIF report

## Figures and Tables

**Table 1:** Hydrogen-bond geometry (Å, °)

*D*—H⋯*A*	*D*—H	H⋯*A*	*D*⋯*A*	*D*—H⋯*A*
C5—H5*A*⋯O3^i^	0.93	2.54	3.449 (7)	165

Symmetry code: (i) 
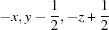
.

**Fig. 1.**
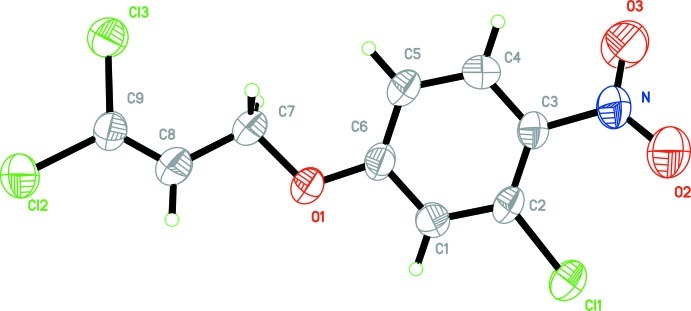
The molecular structure of (I), with the atom-numbering scheme. Displacement ellipsoids are drawn at the 50% probability level.

**Fig. 2.**
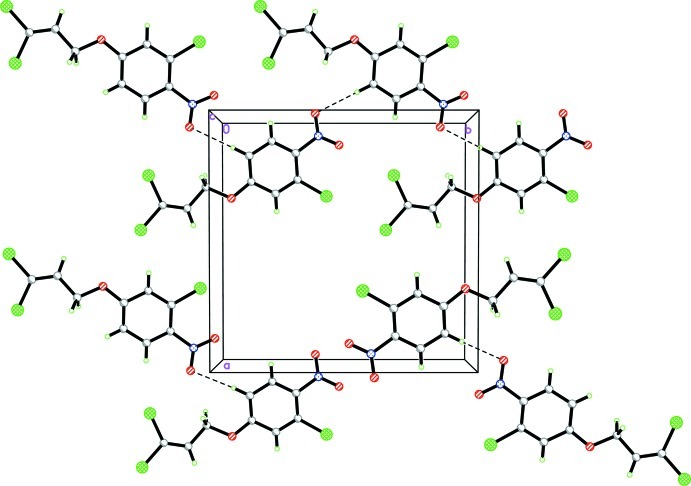
A packing diagram of (I) viewed along the a axis (C-H···O hydrogen bonds are shown as broken lines).

## supplementary crystallographic information

### Comment

The title compound is an important intermediate in the synthesis of phenanthrenes, which can be utilized to synthesize organic semiconductors and conjugated polymers (Walker *et al.*, 2005). These materials are of wide current interest for applications in electronic and optoelectronic devices including light-emitting diodes (Kolosov *et al.*, 2002). We report here the crystal structure of the title compound, (I), which is of interest to us in this field.

The molecular structure of (I) is shown in Fig. 1. There is an intermolecular contact C—H···O in the title compound, forming molecular chains along the *b* axis direction (Table 1, Fig. 2). These molecular chains are linked by weak π—π interactions (Cg1···Cg1^i ^distance = 3.724 (3) Å, Cg1 is the centroid of ring C1-C6, symmetry code: (i) *x*, 5/2 - *y*, -1/2 + *z*) to give a three-dimensional network, which seems to be very effective in the stabilization of the crystal structure.

The dihedral angles between the planes A (atoms C1—C6), B (atoms N/O2/O3), C (atoms C7/C8/H8A/C9/Cl2/Cl3) are: A/B = 16.2 (1)°, A/C = 10.2 (1)°.

### Experimental

The title compound, (I) was prepared by a method reported in literature (Walker *et al.*, 2005). The crystals were obtained by dissolving (I) (0.1 g) in methanol (30 ml) and evaporating the solvent slowly at room temperature for about 8 d.

### Refinement

All H atoms were positioned geometrically and constrained to ride on their parent atoms, with C—H = 0.93 Å for aromatic H and 0.96 Å for alkyl H, respectively. The *U*_iso_(H) = *xU*_eq_(C), where *x* = 1.2 for aromatic H and *x* = 1.5 for other H.

### Crystal data

**Table:**

C_9_H_6_Cl_3_NO_3_	*F*(000) = 568
*M**_r_* = 282.50	*D*_x_ = 1.631 Mg m^−^^3^
Monoclinic, *P*2_1_/*c*	Mo *K*α radiation, λ = 0.71073 Å
Hall symbol: -P 2ybc	Cell parameters from 25 reflections
*a* = 12.476 (3) Å	θ = 10–13°
*b* = 12.775 (3) Å	µ = 0.79 mm^−^^1^
*c* = 7.2230 (14) Å	*T* = 293 K
β = 92.32 (3)°	Block, colourless
*V* = 1150.3 (4) Å^3^	0.30 × 0.20 × 0.10 mm
*Z* = 4	

### Data collection

**Table:**

Enraf–Nonius CAD-4 diffractometer	1414 reflections with *I* > 2σ(*I*)
Radiation source: fine-focus sealed tube	*R*_int_ = 0.023
Graphite monochromator	θ_max_ = 25.4°, θ_min_ = 1.6°
ω/2θ scans	*h* = −15→15
Absorption correction: ψ scan (North *et al.*, 1968)	*k* = −15→0
*T*_min_ = 0.799, *T*_max_ = 0.926	*l* = 0→8
2300 measured reflections	3 standard reflections every 200 reflections
2118 independent reflections	intensity decay: 1%

### Refinement

**Table:**

Refinement on *F*^2^	Primary atom site location: structure-invariant direct methods
Least-squares matrix: full	Secondary atom site location: difference Fourier map
*R*[*F*^2^ > 2σ(*F*^2^)] = 0.066	Hydrogen site location: inferred from neighbouring sites
*wR*(*F*^2^) = 0.183	H-atom parameters constrained
*S* = 1.00	*w* = 1/[σ^2^(*F*_o_^2^) + (0.1*P*)^2^ + 0.7*P*] where *P* = (*F*_o_^2^ + 2*F*_c_^2^)/3
2118 reflections	(Δ/σ)_max_ < 0.001
145 parameters	Δρ_max_ = 0.52 e Å^−^^3^
0 restraints	Δρ_min_ = −0.42 e Å^−^^3^

### Special details

**Table:**

Geometry. All e.s.d.'s (except the e.s.d. in the dihedral angle between two l.s. planes) are estimated using the full covariance matrix. The cell e.s.d.'s are taken into account individually in the estimation of e.s.d.'s in distances, angles and torsion angles; correlations between e.s.d.'s in cell parameters are only used when they are defined by crystal symmetry. An approximate (isotropic) treatment of cell e.s.d.'s is used for estimating e.s.d.'s involving l.s. planes.
Refinement. Refinement of *F*^2^ against ALL reflections. The weighted *R*-factor *wR* and goodness of fit *S* are based on *F*^2^, conventional *R*-factors *R* are based on *F*, with *F* set to zero for negative *F*^2^. The threshold expression of *F*^2^ > σ(*F*^2^) is used only for calculating *R*-factors(gt) *etc*. and is not relevant to the choice of reflections for refinement. *R*-factors based on *F*^2^ are statistically about twice as large as those based on *F*, and *R*- factors based on ALL data will be even larger.

### Fractional atomic coordinates and isotropic or equivalent isotropic displacement parameters (Å^2^)

**Table:**

	*x*	*y*	*z*	*U*_iso_*/*U*_eq_	
N	0.0625 (4)	1.3968 (3)	0.1971 (7)	0.0603 (12)	
Cl1	0.31002 (11)	1.42882 (9)	0.1250 (2)	0.0643 (4)	
C1	0.2996 (4)	1.2216 (4)	0.1245 (7)	0.0468 (11)	
H1A	0.3724	1.2256	0.1021	0.056*	
O1	0.3184 (3)	1.0417 (2)	0.1211 (5)	0.0560 (9)	
Cl2	0.45065 (11)	0.67097 (10)	0.1106 (2)	0.0685 (5)	
C2	0.2415 (4)	1.3117 (3)	0.1436 (7)	0.0452 (11)	
O2	0.1009 (4)	1.4802 (3)	0.2323 (8)	0.1053 (18)	
Cl3	0.22239 (11)	0.70364 (10)	0.1046 (2)	0.0679 (5)	
C3	0.1325 (3)	1.3051 (3)	0.1756 (7)	0.0441 (11)	
O3	−0.0309 (3)	1.3841 (4)	0.1923 (12)	0.157 (3)	
C4	0.0837 (4)	1.2084 (4)	0.1880 (7)	0.0505 (12)	
H4A	0.0105	1.2044	0.2073	0.061*	
C5	0.1433 (4)	1.1170 (4)	0.1718 (7)	0.0496 (12)	
H5A	0.1106	1.0520	0.1837	0.060*	
C6	0.2501 (4)	1.1231 (3)	0.1384 (6)	0.0432 (11)	
C7	0.2718 (4)	0.9380 (3)	0.1296 (8)	0.0585 (14)	
H7A	0.2213	0.9272	0.0256	0.070*	
H7B	0.2340	0.9297	0.2434	0.070*	
C8	0.3611 (4)	0.8612 (4)	0.1230 (7)	0.0542 (13)	
H8A	0.4310	0.8865	0.1258	0.065*	
C9	0.3467 (4)	0.7601 (4)	0.1137 (7)	0.0493 (12)	

### Atomic displacement parameters (Å^2^)

**Table:**

	*U*^11^	*U*^22^	*U*^33^	*U*^12^	*U*^13^	*U*^23^
N	0.055 (3)	0.040 (2)	0.087 (3)	0.0090 (19)	0.005 (2)	−0.007 (2)
Cl1	0.0614 (8)	0.0331 (6)	0.0991 (11)	−0.0085 (5)	0.0112 (7)	−0.0018 (6)
C1	0.042 (2)	0.037 (2)	0.062 (3)	0.0005 (19)	0.013 (2)	0.005 (2)
O1	0.0505 (18)	0.0324 (16)	0.086 (2)	−0.0030 (14)	0.0182 (17)	0.0000 (17)
Cl2	0.0583 (8)	0.0419 (7)	0.1055 (12)	0.0096 (6)	0.0065 (7)	−0.0031 (7)
C2	0.052 (3)	0.026 (2)	0.058 (3)	−0.0048 (19)	0.005 (2)	−0.001 (2)
O2	0.081 (3)	0.042 (2)	0.194 (6)	0.011 (2)	0.022 (3)	−0.013 (3)
Cl3	0.0563 (8)	0.0419 (7)	0.1063 (12)	−0.0047 (6)	0.0121 (7)	−0.0094 (7)
C3	0.044 (2)	0.031 (2)	0.058 (3)	0.0050 (19)	0.002 (2)	0.000 (2)
O3	0.037 (2)	0.069 (3)	0.366 (10)	0.009 (2)	0.019 (4)	−0.050 (5)
C4	0.041 (2)	0.046 (3)	0.065 (3)	−0.004 (2)	0.010 (2)	−0.001 (2)
C5	0.054 (3)	0.030 (2)	0.066 (3)	−0.005 (2)	0.011 (2)	0.003 (2)
C6	0.051 (3)	0.029 (2)	0.050 (3)	0.0001 (19)	0.010 (2)	0.001 (2)
C7	0.048 (3)	0.032 (2)	0.096 (4)	−0.006 (2)	0.010 (3)	−0.002 (3)
C8	0.052 (3)	0.037 (3)	0.074 (3)	−0.005 (2)	0.006 (2)	−0.002 (2)
C9	0.052 (3)	0.037 (3)	0.059 (3)	0.004 (2)	0.008 (2)	0.006 (2)

### Geometric parameters (Å, º)

**Table:**

N—O3	1.176 (6)	Cl3—C9	1.709 (5)
N—O2	1.192 (6)	C3—C4	1.382 (6)
N—C3	1.472 (6)	C4—C5	1.392 (6)
Cl1—C2	1.731 (4)	C4—H4A	0.9300
C1—C2	1.370 (6)	C5—C6	1.367 (6)
C1—C6	1.406 (6)	C5—H5A	0.9300
C1—H1A	0.9300	C7—C8	1.487 (6)
O1—C6	1.353 (5)	C7—H7A	0.9700
O1—C7	1.449 (5)	C7—H7B	0.9700
Cl2—C9	1.727 (5)	C8—C9	1.304 (7)
C2—C3	1.392 (6)	C8—H8A	0.9300
			
O3—N—O2	121.2 (5)	C6—C5—H5A	120.2
O3—N—C3	118.6 (4)	C4—C5—H5A	120.2
O2—N—C3	119.9 (4)	O1—C6—C5	126.4 (4)
C2—C1—C6	120.6 (4)	O1—C6—C1	113.6 (4)
C2—C1—H1A	119.7	C5—C6—C1	119.9 (4)
C6—C1—H1A	119.7	O1—C7—C8	107.5 (4)
C6—O1—C7	116.4 (4)	O1—C7—H7A	110.2
C1—C2—C3	119.4 (4)	C8—C7—H7A	110.2
C1—C2—Cl1	117.0 (4)	O1—C7—H7B	110.2
C3—C2—Cl1	123.6 (3)	C8—C7—H7B	110.2
C4—C3—C2	120.1 (4)	H7A—C7—H7B	108.5
C4—C3—N	116.1 (4)	C9—C8—C7	123.6 (5)
C2—C3—N	123.9 (4)	C9—C8—H8A	118.2
C3—C4—C5	120.4 (4)	C7—C8—H8A	118.2
C3—C4—H4A	119.8	C8—C9—Cl3	122.9 (4)
C5—C4—H4A	119.8	C8—C9—Cl2	123.4 (4)
C6—C5—C4	119.7 (4)	Cl3—C9—Cl2	113.7 (3)
			
C6—C1—C2—C3	−0.5 (7)	C3—C4—C5—C6	−1.8 (7)
C6—C1—C2—Cl1	179.8 (4)	C7—O1—C6—C5	3.3 (7)
C1—C2—C3—C4	0.0 (7)	C7—O1—C6—C1	−178.5 (4)
Cl1—C2—C3—C4	179.8 (4)	C4—C5—C6—O1	179.4 (5)
C1—C2—C3—N	−179.6 (5)	C4—C5—C6—C1	1.3 (7)
Cl1—C2—C3—N	0.1 (7)	C2—C1—C6—O1	−178.4 (4)
O3—N—C3—C4	−12.3 (8)	C2—C1—C6—C5	−0.2 (7)
O2—N—C3—C4	162.1 (5)	C6—O1—C7—C8	−175.5 (4)
O3—N—C3—C2	167.3 (6)	O1—C7—C8—C9	−174.5 (5)
O2—N—C3—C2	−18.2 (8)	C7—C8—C9—Cl3	0.9 (8)
C2—C3—C4—C5	1.1 (7)	C7—C8—C9—Cl2	−178.7 (4)
N—C3—C4—C5	−179.2 (5)		

### Hydrogen-bond geometry (Å, º)

**Table:**

*D*—H···*A*	*D*—H	H···*A*	*D*···*A*	*D*—H···*A*
C5—H5*A*···O3^i^	0.93	2.54	3.449 (7)	165

Symmetry code: (i) −*x*, *y*−1/2, −*z*+1/2.
